# The Emory‐Sage‐SGC‐JAX TREAT‐AD Center: Developing Resources for Alzheimer’s Disease Novel Targets

**DOI:** 10.1002/alz70859_103791

**Published:** 2025-12-25

**Authors:** Karina Leal, Elizabeth L Zoeller, Gregory A. Cary, Jesse C Wiley, Yuhong Du, Levon Halabelian, Alison D. Axtman, Ranjita Betarbet, Gregory W Carter, Haian Fu, Frank Longo, Stacey J Sukoff Rizzo, Aled M Edwards, Allan I. Levey, The Emory‐Sage‐SGC‐JAX TREAT‐AD Center

**Affiliations:** ^1^ Sage Bionetworks, Seattle, WA USA; ^2^ Emory University, Atlanta, GA USA; ^3^ The Jackson Laboratory, Bar Harbor, ME USA; ^4^ University of Toronto, Toronto, ON Canada; ^5^ University of North Carolina at Chapel Hill, Chapel Hill, NC USA; ^6^ Stanford University, Stanford, CA USA; ^7^ University of Pittsburgh School of Medicine, Pittsburgh, PA USA; ^8^ Structural Genomics Consortium, Toronto, ON Canada

## Abstract

**Background:**

Alzheimer’s Disease (AD) is a debilitating neurodegenerative disorder affecting an estimated 55 million people world‐wide. With no clear understanding of disease mechanism, hypothesis‐based AD drug discovery has proven to be a high‐risk endeavor. The TREAT‐AD Consortium was initiated to improve, diversify, and reinvigorate the AD drug development pipeline by accelerating the characterization and experimental validation of next generation therapeutic targets. To this end, the Emory‐Sage‐SGC‐JAX TREAT‐AD Center adopted the concept of a target enabling package (TEP) to bridge the gap between target discovery and new therapeutics and to stimulate drug discovery by catalyzing investigation across the field for potential therapeutic pathways. Our aim is to allow for rapid exploration of emerging therapeutic hypotheses and novel AD therapeutic targets, including those emanating from the NIA‐funded target discovery programs in AD, and initiate early‐stage drug discovery campaigns against the enabled targets.

**Method:**

Given the demonstrated heterogeneity of AD in biological and genetic components, it is critical to identify new therapeutic approaches. Our Center developed an iterative target prioritization pipeline to score candidate proteins and organize groups of co‐functional proteins into standardized therapeutic hypotheses for validation. Nominated targets were evaluated by calculating an unbiased target AD risk score that is then mapped to 19 biological domains that describe and codify the different processes that are dysregulated in AD. Targets that meet criteria for development are evaluated to identify a set of experimental reagents necessary for hypothesis testing.

**Result:**

Our center has developed TEPs for more than 50 understudied targets. For each prioritized target, a TEP may include bioinformatic analysis, expression constructs, purified protein and methods, validated knockout cell lines, antibody validation, assay development, crystal structures, screening, and probe development. All reagents are developed to meet established quality criteria. Advanced targets include DHX58, SYK, DDX1, SDC4, CAPN1, ARHGEF2, and members of ‘Module 42’ including SMOC1.

**Conclusion:**

All data, protocols, reagent sets, and chemical tools are made widely available on the AD Knowledge Portal with no intellectual property claims. Target Risk Scores and biodomains are accessible through Agora. For more information and the full target portfolio see www.treatad.org.

